# Effect of whole course seamless nursing mode on patients with chronic infectious wounds

**DOI:** 10.4314/ahs.v23i2.25

**Published:** 2023-06

**Authors:** Yun Peng, Chenlu Gao, Yan Sun

**Affiliations:** Qingdao Central Hospital, 127 Siliu South Road, Qingdao 266000, Shandong Province, China

**Keywords:** Chronic infectious wound, whole course seamless nursing, quality of life

## Abstract

**Background:**

Chronic infectious wounds seriously affect patients' quality of life.

**Aim:**

To assess the effect of whole course seamless nursing mode on patients with chronic infectious wounds.

**Methodology:**

One hundred patients treated between January 2019 and December 2020 were randomly divided into control and observation groups (n=50) that were given routine nursing and whole course seamless nursing, respectively. Their pain score, comfort score, wound healing time, wound healing effect, psychological state scores, sleep indices, quality-of-life scores and degree of satisfaction with nursing were compared.

**Results:**

Observation group had lower pain score and higher comfort score than those of control group after nursing (P<0.05). Compared with control group, observation group had shorter wound healing time and higher grade-A wound healing rate (P<0.05). The SDS and SAS scores of observation group were lower than those of control group (P<0.05). Observation group also had significantly shorter sleep latency, longer actual sleep time, lower PSQI score, as well as higher quality-of-life score and overall satisfaction rate than those of control group (P<0.05).

**Conclusion:**

For patients with chronic infectious wounds, whole course seamless nursing effectively relieves wound pain, facilitates wound healing, improves comfort, psychological state and sleep status, and makes them more satisfied.

## Introduction

Chronic infectious wounds refer to the wound infection induced by the invasion of pathogenic microorganisms after injury, leading to delayed wound healing or even unhealed wounds and seriously affecting patients' quality of life^1^-^3^. Considering the slow healing of chronic infectious wounds, it is necessary to perform reasonable nursing measures to better facilitate wound healing^4^. Whole course seamless nursing is a new nursing mode based on the combination of whole course nursing and seamless nursing, which advocates active intervention against the “seam” in the process of nursing. In this study, 100 patients with chronic infectious wounds treated between January 2019 and December 2020 were selected to assess the effect of whole course seamless nursing on patients with chronic infectious wounds.

## Materials and methods

### Subjects

Recruitment was carried out by educating patients with chronic infectious wounds regarding the purpose and significance of this study, and then they voluntarily participated in the study. We conducted a prospective case-control study and included 100 patients with chronic infectious wounds treated between January 2019 and December 2020. They were divided into control and observation groups (n=50) using a random number table. This study has been approved by the hospital's ethics committee, and written informed consents have been obtained from all patients and their family members.

### Interventions

The control group was subjected to routine nursing. Specifically, the wound was cleaned and disinfected and the dressing was changed regularly according to the doctor's advice to ensure that the dressing was clean and dry until the wound healed.

Patients in observation group were given whole course seamless nursing. A whole course seamless nursing group was set up, and a nursing plan was formulated according to the specific conditions of the patients. The specific measures were as follows:

(1) Health education: After admission, health education was conducted for the patients. Pictures and videos were utilized to explain the causes, treatment methods and relevant precautions of chronic infectious wounds in detail for patients and their families. Then the patients' queries were listened to and answered. In the case of complication with diabetes, hypertension or other chronic basic diseases, the patients were guided to take hypoglycemic drugs or antihypertensive drugs according to the doctor's advice, so as to control blood glucose and blood pressure.

2) Psychological nursing: The basic information of the patients was collected. The nursing staff communicated with the patients, and understood the patients' psychological dynamics by listening carefully to them. In addition, the nursing staff analysed the causes of negative emotions together with the patients, based on which they appeased and enlightened the patients, and illustrate cases where chronic infectious wound was successfully cured for the patients, so as to give the patients positive hints, and encouraged the patients to build up confidence.

(3) Wound nursing: The patients' wound was cleaned, and a silicone drainage tube was placed at the wound as prescribed by the doctor. After the drainage tube was fixed with medical adhesive plaster, the wound was covered with a layer of aseptic biological semi-permeable membrane, which was fit to the silicone drainage tube, leaving no gap. Next, a small opening was trimmed on the aseptic biological semi-permeable membrane, and covered with a layer of aseptic Vaseline gauze. After the drainage tube was wrapped, the second layer of aseptic biological semi-permeable membrane and the second layer of aseptic Vaseline gauze were applied to form a closed negative pressure environment. Subsequently, the drainage tube was connected to a device of negative pressure suction. The negative pressure sealing materials were replaced every other week until the wound healed.

(4) Discharge nursing: On the discharge day, the patients and their family members were informed of the cautions needing attention after discharge, and instructed the patients to regularly return to the hospital for re-examination. After discharge, the wound healing status of the patients were regularly followed up by telephone (once a week), and the patients were guided to have reasonable diet and maintain clean and ventilated home environment. In case of no granulation growth at the wound, it was necessary for the patients to return to the hospital for treatment.

### Observation indices

The pain score, comfort score, wound healing time, wound healing effect, psychological state scores, sleep indices, quality-of-life scores and degree of satisfaction with nursing of the two groups were compared.

Pain score: The pain degree was evaluated by using the visual analogue scale (VAS). The score ranged 0-10 points, and 0 indicated no pain. The higher the score, the severer the pain.

**Comfort score:** The comfort level was assessed with the General Comfort Questionnaire (GCQ) including 28 items (each item: 1-4 points; total score: 28-112 points). A lower score means less comfort.

Based on the wound healing status, the wound healing effect could be divided into the following grades: 1) Grade A: the epidermis of the wound healed completely without redness and swelling, 2) Grade B: the epidermis of the wound healed basically with slight redness and swelling, and 3) Grade C: the epidermis of the wound did not heal and there was obvious redness and swelling.

**Psychological state scores:** The psychological state scores included anxiety score and depression score. The anxiety degree was evaluated by the Self-rating Anxiety Scale (SAS) (total score: 100 points). According to the Chinese norm, the cut-off value was set at 50 points, and a lower score means milder anxiety. The depression degree was assessed with the Self-rating Depression Scale (SDS) (total score: 100 points). According to the Chinese norm, the cut-off value was set at 53 points, and a lower score suggests milder depression.

Sleep indices included sleep latency, actual sleep time and sleep quality score. The sleep quality was assessed with the Pittsburgh Sleep Quality Index (PSQI) including 7 items (each item: 0-3 points; total score: 0-21 points). A lower score indicates better sleep quality.

**Quality-of-life scores:** Four domains (0-100 points), i.e., physical health, psychological health, environment and social relationships, were assessed by using the World Health Organization Quality of Life Scale Brief Version (WHOQOL-BREF). A lower score indicates poorer quality of life.

**Degree of satisfaction with nursing:** The satisfaction degree was investigated with a self-made questionnaire. The highest score was 100 points (<60 points: unsatisfactory; 60-80 points: generally satisfactory; >80 points: highly satisfactory). The total satisfaction rate was calculated as follows: Overall satisfaction rate = general satisfaction rate + high satisfaction rate.

### Statistical analysis

All data were statistically analysed by SPSS 22.0 software. The categorical variables were represented as percentage and analysed using the chi-square test. The continuous numerical variables were represented as mean ± standard deviation (-x ± s). The independent t test was used for comparison between groups at the same time point, and the paired t test was used for comparison within groups before and after nursing. P<0.05 indicated that a difference was statistically significant.

## Results

### Socio-demographic characteristics of the study population

The control group included 27 males and 23 females aged 20-65 years old, (43.25±12.65) years on average. There were 22 cases of ASA grade II and 28 cases of grade III. The observation group consisted of 29 males and 21 females aged 20-64 years old, (42.91±12.73) years on average. There were 23 cases of ASA grade II and 27 cases of grade III. The two groups had similar gender ratio, age and ASA grade (P>0.05). The socio-demographic characteristics are listed in Table 1.

**Table 1:** Socio-demographic characteristics of study population

Item	Observation group	Control group	P (95%CI)
Age	42.91±12.73	43.25±12.65	0.894 (0.821-1.247) ^a^
Gender			0.687 (0.782-1.034) ^b^
Male	29	27	
Female	21	23	
ASA grade			0.840 (0.672-1.125) ^b^
II	23	22	
III	27	28	

a Independent t test

b chi-square test

### Pain and comfort scores

Compared with before nursing, pain score was significantly lower in the two groups after nursing, especially in observation group (P<0.05). After nursing, both groups, especially control group, had significantly increased comfort scores (P<0.05) (Table 2).

**Table 2:** Pain and comfort scores (x̅ ± s, point)

Group	Time	VAS score	GCQ score
Control (n=50)	Before nursing	4.53±1.02	74.82±7.51
	After nursing	4.46±1.05^#^	75.09±7.46^#^
P (95%CI) ^a^		0.002 (1.092-1.892)	0.012 (1.234-2.932)
Observation (n=50)	Before nursing	3.29±0.83	85.24±9.02
	After nursing	2.47±0.74^#^*	96.43±9.87^#^*
P (95%CI) ^a^		<0.001 (1.293-2.345)	<0.001 (1.763-3.421)
P (intergroup comparison after nursing) (95%CI) ^b^		<0.001 (1.022-1.982)	<0.001 (1.562-2.432)

# P<0.05 *vs*. before nursing

* P<0.05 *vs*. control group

a Paired t test

b independent t test

### Wound healing results

Compared with control group, observation group had shorter wound healing time, and higher grade-A wound healing rate (P<0.05) (Table 3).

**Table 3:** Wound healing results

Group	n	Wound healing time (d)	Wound healing grade (n (%))		
			
			Grade A	Grade B	Grade C
Control	50	12.63±1.52	33 (66.00%)	12 (24.00%)	5 (10.00%)
Observation	50	10.91±1.37*	42 (84.00%) *	7 (14.00%)	1 (2.00%)
P (95%CI)		<0.001 (1.782-3.023) ^a^	0.028 (1.129-1.982) ^b^		

* P<0.05 *vs*. control group

a Independent t test

b chi-square test

### Psychological state scores

Compared with before nursing, SDS and SAS scores significantly decreased in the two groups after nursing, being more obvious in observation group (P<0.05) (Table 4).

**Table 4:** Psychological state scores (χ̅ ± s, point)

Group	Time	SAS score	SDS score
Control (n=50)	Before nursing	54.21±6.90	55.28±6.61
	After nursing	43.74±2.93^#^	45.16±3.07^#^
P (95%CI) ^a^		<0.001 (1.273-2.734)	<0.001 (1.027-1.925)
Observation (n=50)	Before nursing	54.31±6.82	55.38±6.50
	After nursing	40.80±2.74^#^*	42.05±2.81^#^*
P (95%CI) ^a^		<0.001 (1.125-2.093)	<0.001 (1.276-2.303)
P (intergroup comparison after nursing) (95%CI) ^b^		<0.001 (1.220-3.242)	<0.001 (1.329-4.365)

# P<0.05 *vs*. before nursing

* P<0.05 *vs*. control group

a Paired t test

b independent t test

### Sleep indices

After nursing, both groups, particularly observation group, had significantly improved actual sleep time, sleep latency and PSQI score compared with those before nursing (P<0.05) (Table 5).

**Table 5:** Sleep indices (x̅ ± s)

Group	Time	Sleep latency (min)	Actual sleep time (h)	PSQI score (point)
Control (n=50)	Before nursing	64.45±12.71	4.16±1.24	15.20±2.35
	After nursing	42.63±8.75^#^	6.89±1.30^#^	11.26±1.58^#^
P (95%CT) ^a^		<0.001 (1.118-1.874)	<0.001 (1.014-1.911)	<0.001 (1.237-2.984)
Observation (n=50)	Before nursing	64.70±12.58	4.14±1.25	15.27±2.32
	After nursing	33.79±7.94^#^*	8.32±1.43^#^*	9.60±1.41^#^*
P (95%CT) ^a^		<0.001 (1.782-2.985)	<0.001 (1.432-2.235)	<0.001 (1.562-1.824)
P (intergroup comparison after nursing) (95%CI) ^b^		<0.001 (2.113-4.325)	<0.001 (1.092-3.228)	<0.001 (1.162-2.946)

# P<0.05 *vs*. before nursing

* P<0.05 *vs*. control group

a Paired t test

b independent t test

### Quality-of-life scores

After nursing, the two groups had significantly higher quality-of-life scores than those before nursing, especially in observation group (P<0.05) (Table 6).

**Table 6:** Quality-of-life scores (x̅ ± s, point)

Group	Time	Physical health	Psychological health	Environment	Social relationship
Control (n=50)	Before nursing	70.81±5.20	70.23±5.14	70.34±5.01	70.48±5.09
	After nursing	77.34±6.42^#^	77.87±6.17^#^	76.46±5.23^#^	76.73±5.40^#^
P (95%CI) ^a^		<0.001 (1.923-3.422)	<0.001 (2.032-4.203)	<0.001 (1.284-3.235)	<0.001 (1.426-3.294)
Observation (n=50)	Before nursing	70.96±5.17	70.45±5.13	70.48±5.09	70.61±5.16
	After nursing	84.05±6.59^#^*	84.39±6.28^#^*	83.47±6.83^#^*	83.62±5.71^#^*
P (95%CI) ^a^		<0.001 (1.123-2.092)	<0.001 (1.465-2.742)	<0.001 (1.338-3.424)	<0.001 (1.273-3.684)
P (intergroup comparison after nursing) (95%CI) ^b^		<0.001 (1.832-3.224)	<0.001 (1.294-1.723)	<0.001 (1.398-2.033)	<0.001 (1.409-2.423)

# P<0.05 *vs*. before nursing

* P<0.05 *vs*. control group

a Paired t test

b independent t test

### Nursing satisfaction rates

The overall nursing satisfaction rate of observation group exceeded that of control group (98.00% vs. 84.00%, P<0.05) (Table 7).

**Table 7:** Nursing satisfaction rates (n (%))

Group	n	High satisfaction	General satisfaction	No satisfaction	Overall satisfaction
Control	50	24 (48.00%)	18 (36.00%)	8 (16.00%)	42 (84.00%)
Observation	50	30 (60.00%)	19 (38.00%)	1 (2.00%)	49 (98.00%)*
P (95%CI) ^a^		<0.001 (1.021-1.723)			

* P<0.05 *vs*. control group.

a Chi-square test.

## Discussion

Chronic infectious wounds refer to wounds (due to various factors) infected with microorganisms during the healing stage, which are common in surgery^5^-^7^. The recovery of chronic infectious wounds is intractable, which is prone to delayed wound healing or even unhealed wounds, seriously affecting the daily life of patients^8^-^10^. In addition, long-term unhealed wounds will cause severe pain, affecting the psychological state and sleep status of patients^11^,^12^.

It is clinically recommended to perform active therapies for chronic infectious wounds, such as dressing change treatment and vacuum sealing drainage^13^. However, insufficient knowledge and poor psychological state of patients may cause poor therapeutic effects^14^-^16^. Therefore, it is crucial to perform reasonable nursing interventions for such patients. Routine nursing measures mainly include regular wound cleaning and dressing change, but lack insufficient attention to psychological condition, which makes patients unsatisfied with nursing services. In recent years, whole course seamless nursing mode has been applied gradually in clinical practice. The core concept of this mode is “seamless”, which aims to eliminate all seams during the nursing process, thus removing potential nursing hazards, avoiding nursing risks, and providing better nursing services for patients. From the day of admission to discharge from hospital or even later, whole course seamless nursing mode offers comprehensive and continuous nursing interventions, with wide ranges of time and space scales, which can make up for the shortcomings of routine nursing measures such as one-sidedness and lack of continuousness. In the present study, whole course seamless nursing mode was adopted in observation group, through optimizing the details of nursing in patients with chronic infectious wounds from the day of admission to discharge from hospital to achieve the goal of “seamless nursing”, thus improving the quality of nursing services. The results of this study revealed that observation group had lower pain score, higher grade-A wound healing rate and comfort score, and shorter wound healing time than those of control group after nursing (P<0.05), indicating that whole course seamless nursing can effectively reduce physical discomfort and speed up wound healing. After nursing, observation group had significantly lower SAS, SDS and PSQI scores than those of control group, and higher quality-of-life scores (P<0.05), demonstrating that whole course seamless nursing can reduce the patient's physical discomfort and the resulting adverse effects on their psychology, daily life and sleep. Besides, observation group had higher overall satisfaction rate than that of control group (98.00% vs. 84.00%, P<0.05), displaying that whole course seamless nursing, serving as a more detailed, comprehensive and targeted nursing strategy, can better meet the patient's actual requirement, thus improving the evaluation of nursing services by patients.

In conclusion, the application of whole course seamless nursing in patients with chronic infectious wounds can effectively relieve wound pain, improve comfort, facilitate wound healing, ameliorate the psychological state, sleep and quality of life, and raise the degree of satisfaction with nursing service. Regardless, this study is still limited. This is a single study with a small sample size, so the results may be biased. Further multicenter studies with larger sample sizes are in need to validate our conclusion.
